# Supplementary material for: MS1Connect: a mass spectrometry run similarity measure
Source: Bioinformatics. 2023 Jan 25;39(2):btad058. doi: 10.1093/bioinformatics/btad058 (PMC9913042; doi:10.1093/bioinformatics/btad058)
Supplement: btad058_Supplementary_Data [file btad058_supplementary_data.zip › btad058_Supplementary_Data/ms1Paper-supplement.pdf]

Supplement to "MS1Connect: a mass spectrometry run similarity measure"

Andy Lin[1], Brooke L. Deatherage Kaiser[1], Janine R. Hutchison[1], Jeffrey A. Bilmes[2], and William Stafford Noble[*3,4]

[1]Chemical and Biological Signatures, Pacific Northwest National Laboratory, Richland, WA, USA
[2]Department of Electrical and Computer Engineering, University of Washington, Seattle, WA, USA
[3]Department of Genome Sciences, University of Washington, Seattle, WA, USA
[4]Paul G. Allen School for Computer Science and Engineering, University of Washington, Seattle, WA, USA

1 Approach

1.1 Representation of a mass spectrometry run

There are four values that are returned by pyOpenMS: m/z, intensity, charge, and retention time (in seconds). Prior to analysis, we replaced two of the three values with normalized versions thereof. For the retention time normalization, we first selected the top N most intense MS1 features to represent an entire run, where N is a hyperparameter of MS1Connect. Then, we normalized the retention times of the N features on a scale of zero to one based on the proportion of total ion current detected up to a given time. In addition, we removed MS1 features with a normalized retention time less than 0.05 and greater than 0.95. Finally, we scaled the intensities to range from zero to one by dividing by the maximum intensity among all MS1 features in the given run.

1.2 Selecting the best matching

As previously noted, computing a maximization over an exponential number of edge subsets is generally intractable. However, our particular setup allows for an efficient computational of an approximate solution. First, we note that $M(A) = \sum_{e_1,e_2 \in A} m(e_1, e_2)$ is itself a function over subsets of edges, and that the function sums over the submatrix associated with edge set A. Given two subsets A, B, since it is the case that $m(e_1, e_2) \geq 0$ for all e_1, e_2, this function is said to be *supermodular* because it has the property of that $M(A) + M(B) \leq M(A \cup B) + M(A \cap B)$.

Second, we see that $\mathcal{E}_U$ and $\mathcal{E}_V$ correspond to a set of subsets of edges that have certain critical properties:

1. $\emptyset \in \mathcal{E}_U$,
2. if $B \in \mathcal{E}_U$ and $A \subseteq B$ then $A \in \mathcal{E}_U$, and
3. if $A, B \in \mathcal{E}_U$ with $|A| < |B|$, then $\exists b \in B \setminus A$ such that $A + b \in \mathcal{E}_U$.

*Corresponding author: Box 355065, Foege Building, S220B, 3720 15th Ave NE, Seattle, WA 98195-5065, william-noble@uw.edu

These three properties define a matroid.[1,2] In fact, $\mathcal{E}_U$ and $\mathcal{E}_V$ are a particular kind of matroid, called a *partition matroid*. A partition matroid says that we partition the edges E into disjoint blocks. For A to be independent in the matroid means that A must not intersect each block by more than a certain limit. In the present case, the blocks of edges are defined by the edges incident to each of the nodes $u \in U$ and $v \in V$. Thus, the constraint $A \in (\mathcal{E}_U \cap \mathcal{E}_V)$ means that for A to be feasible, it must simultaneously be a member of the independent sets of two partition matroids $\mathcal{E}_U$ and $\mathcal{E}_V$. Hence, to produce our run-pair score, we must solve an instance of supermodular maximization subject to two matroid constraints.

Ordinarily, computing the solution to such a problem is inapproximable, because supermodular maximization subject to even simple constraints in general is very hard and even hard to approximate.[3,4] However, recent work has shown that if the supermodular function exhibits certain properties, then an approximation algorithm is possible using a greedy procedure.[5] Ordinarily, the greedy algorithm can do arbitrarily poorly when the function is supermodular (see[4] for a simple example). However, when the function has limited curvature, an approximation is possible. We define the supermodular curvature as $\kappa^f = 1 - \min_{e \in E} f(e)/f(e \backslash E)$. It has been showed that $0 \leq \kappa^f \leq 1$ and if $\kappa^f = 1$, then constrained supermodular maximization is indeed inapproximable.[5] However, if $\kappa^f < 1$, then the greedy algorithm achieves an approximation bound of $1 - \kappa^f$, which means that $f(\tilde{S}) \geq (1 - \kappa^f) f(S^*)$ where $\tilde{S}$ is the solution provided by the greedy algorithm, and S^* is the optimal solution.

The greedy algorithm is fairly simple. We start with $S \leftarrow \emptyset$ and we then repeat the step

$$S \leftarrow S \cup \text{argmax}_{e \in E \backslash S} f(S + e)$$

until the constraints are no longer satisfied. After this procedure finishes, we multiply in the de-normalization term $(\sum_{i=1}^{4} \lambda_i M_i(E))$ as a post-processing step. This algorithm runs quickly and scalably even with very large sets $|E|$, and we therefore use this procedure for computing the score in Equation 2.

The curvature of f is very dependent on the matrix M. It turns out that as long as the diagonal entries of M are non-zero, then $\kappa^g < 1$. In Supplementary Figure 4, we show that our best results correspond to the case where the diagonal is non-zero, and this happens as long as $\lambda_4 < 1$ in Equation (4), and thus $\kappa^g < 1$. These observations offer mathematical justification for the performance of our algorithm.

1.3 Description of each MS1Connect term

The first term M_1 counts the number of edges selected in a solution: $M_1(A) = |A|$. Thus, this term favors solutions that contain a large number of edges. Runs that are similar will share many of the same peptides. As a result, these peptides will be linked by edges, thereby generating many edges.

The second term sums the product of the two intensities associated with each edge found in a selected matching, $M_2(A) = \sum_{e \in A} I(u(e)) I(v(e))$, where $I(u)$ in the intensity of feature u and $I(v)$ in the intensity of feature v. This term favors solutions that select edges with high intensity values. The intensity of an MS1 feature produced by a peptide is a function of the sequence and abundance of that peptide. Runs that are similar to each other should have similar peptide expression levels. By multiplying the two intensities together, we focus on the most intense peptide features.

The third term sums the negative exponent of the absolute difference of the normalized retention time shift of all the selected edges: $M_3(A) = \sum_{e \in A} \exp(-\alpha |s(e)|)$, where α is a hyperparameter that determines how quickly the exponential function decreases and $s(e)$ is the normalized retention time shift between u and v: $s(e) = t(v(e)) - t(u(e))$, where $t(u)$ and $t(v)$ are the normalized retention times of MS1 features u and v, respectively. This term favors edges that have small normalized retention time shifts. Such edges are better because they are more likely to link the same peptide feature. This is because we expect the time it takes for peptides to elute off the column, in similar chromatography conditions, to be similar from one run to the next.

Finally, the fourth term scores pairs of edges rather than a single edge. This term favors solutions that contain many similar pairs of edges. We say a pair of edges are similar to each other when they have similar normalized retention time shifts and the normalized retention times of the MS1 features, in the same run, are similar to each other. We define the term as

$$M_4(A) = \sum_{e_1, e_2 \in A} \exp(-\beta |s(e_1) - s(e_2)|) \exp(-\gamma |t(v(e_1)) - t(v(e_2))|), \tag{1}$$

where β and γ are hyperparameters. The first exponent is large when the normalized retention time shifts of the two edges are similar. On the other hand, the second exponent is large when pairs of MS1 features in the same run have similar normalized retention times. Altogether, this term scores edge pair similarity while allowing for systematic retention time shifts between two different mass spectrometry runs. Such shifts are common, reflecting differences in chromatography conditions between runs. Note that M_4 would perform well if the retention times of two runs are optimally aligned with each other because aligned runs would result in systemically small retention time shifts. In addition, for efficiency we set M_4 to zero when $|s(e_1) - s(e_2)| > 0.01$ or when $|t(v(e_1)) - t(v(e_2))| > 0.01$.

2 Methods

2.1 Baseline similarity measures

In order to ensure that our objective obtained sensible results, we compared MS1Connect against several baseline similarity measures. One set of baseline similarity measures are based on binning the MS1 features and computing scalar products. The other set counts the number of MS1 features within a m/z and normalized retention time tolerance.

For the binned baseline, we represent the two runs, X and Y, as matrices with dimensions m and n, where m is the number of m/z bins and n is the number of retention time bins. The value in each cell is the number of MS1 features whose discretized m/z and retention time fall into that bin. Both δ_2, the m/z bin width, and n, the number of retention time bins, are hyperparameters. More formally, the binned baseline scores are defined as

$$S_{X,Y} = \sum_{i=0}^{m} \sum_{j=0}^{n} G(X_{i,j}, Y_{i,j}), \quad (2)$$

where X and Y are the matrix representations of the proteomics runs and G is the product, maximum, or minimum of the two input values. Alternatively, G could be defined as $G(S,T) = \mathbb{1}_{\{S \geq 1\}} \mathbb{1}_{\{T \geq 1\}}$, where $\mathbb{1}$ is the indicator function. Finally, we considered baselines that do not consider retention time ($n = 1$) separately from baselines that do consider retention time ($n \geq 2$), for a total of eight different baselines.

For the continuous baseline, we represented a pair of runs as a bipartite graph, just like in the formulation of MS1Connect. The score for this baseline is the total number of edges connecting the two runs. Note that the difference between this baseline and the M_1 term of MS1Connect is that the baseline measure does not have the degree constraint. We also considered the baseline that does not consider retention time (normalized RT threshold$= 1$) separately from the baseline that does consider retention time (normalized RT threshold < 1).

2.2 Hyperparameter search

MS1Connect ($M_1 - M_4$) has nine different hyperparameters (Table 1). Two of them affect the generation of the bipartite graph while the remaining seven hyperparameters affect the scoring of a specific selected edge set. The first hyperparameter, N, is the maximum number of MS1 features used, in each run, to generate the bipartite graph. The next hyperparameter δ_1 is the m/z threshold (in ppm) used for connecting two MS1 features. Each of the four λ hyperparameters are used to weight each of the four terms in the objective function. Finally, α, β, and γ affect the rate at which the exponential functions decrease in M_3 and M_4.

To determine the best performing hyperparameter set, as measured by QAP, we sampled the hyperparameter space using a random grid search. In a random grid search, the range and values of each hyperparameter is predetermined. Then, for each instance of a hyperparameter set, random values are selected from the grid. Our hyperparameter grid consists of 954,976 possible sets of hyperparameters (Supplemental Table 1). The number of MS1 features used in the bipartite graph generation ranged from 1,000 to 4,000 with two fold-change increments. The m/z tolerance ranged from 1 ppm to 16 ppm with four-fold change increments. All four λ hyperparameters were allowed to range between zero and one, inclusive, with linear increments of 0.1, subject to the constraint that $\sum_1^4 \lambda_i = 1.0$. Finally, the hyperparameters α, β, γ ranged from 10^{-6} to 1.0, with 10-fold change increments. We evaluated a total of 1,716 different randomly chosen hyperparameter sets.

hyperparameter	possible values
# of MS1 features	1000, 2000, 4000, 8000
m/z tolerance (ppm)	1, 4, 16, 64
α	10^{-6}, 10^{-5},..., 1.0
β	10^{-6}, 10^{-5},..., 1.0
γ	10^{-6}, 10^{-5},..., 1.0
λ_1	0.0, 0.1,..., 1.0
λ_2	0.0, 0.1,..., 1.0
λ_3	0.0, 0.1,..., 1.0
λ_4	0.0, 0.1,..., 1.0

Supplemental Table 1: **MS1Connect hyperparameter search grid.** Table of the grid that was searched during the hyperparameter search. We conducted an exhaustive hyperparameter search for each version of MS1Connect that only considered a single term, which yielded 784 hyperparameter sets. In addition, we evaluated 1,716 randomly chosen sets of hyperparameters. Altogether, we evaluated 2,500 out of the 954,976 possible hyperparameter sets.

Method	# MS1 features	m/z tolerance (ppm)	λ_1	λ_2	λ_3	λ_4	α	β	γ
MS1Connect ($M_1 - M_4$)	4000	4	0.0	0.1	0.0	0.9	0.1	10^{-5}	1.0
MS1Connect (M_1 only)	1000	4	1.0	0.0	0.0	0.0	NA	NA	NA
MS1Connect (M_2 only)	500	16	0.0	1.0	0.0	0.0	NA	NA	NA
MS1Connect (M_3 only)	1000	4	0.0	0.0	1.0	0.0	10^{-5}	NA	NA
MS1Connect (M_4 only)*	4000	4	0.0	0.0	0.0	1.0	NA	1.0	0.001

Supplemental Table 2: **Best performing MS1Connect hyperparameters.** The best performing set of hyperparameters for each MS1Connect method. *Six different hyperparameterizations of MS1Connect (M4) have the best performance. Specifically, the best performance occurs when β and γ are 0.1 and 10^{-5}, 0.1 and 10^{-6}, 1.0 and 10^{-3}, 1.0 and 10^{-4}, 1.0 and 10^{-5}, or 1.0 and 10^{-6} respectively.

In addition to a hyperparameter search for MS1Connect ($M_1 - M_4$), we conducted an exhaustive hyperparameter search for each of the four versions of MS1Connect that only used a single term. Each version used the same hyperparameter grid as MS1Connect ($M_1 - M_4$) except that we added two additional values (250 and 500) to the number of MS1 features for the variants of MS1Connect that only use M_1, M_2, or M_3. We note that not all versions of MS1Connect use all the previously described hyperparameters. For example, the only relevant hyperparameters in MS1Connect (M_1 only) are number of MS1 features and m/z tolerance. The best performing set of hyperparameters for each method can be found in Supplemental Table 2. Note that MS1Connect (M_4 only) has six different sets of hyperparameters that yield the best performance.

Finally, we performed an exhaustive hyperparameter search for our eight binned baselines and two continuous baseline (Supplemental Table 3). The binned baselines have three different hyperparameters: number of MS1 features, m/z bin width δ_2, and number n of retention time bins. The hyperparameter grid allowed δ_2 to range from 0.0025 to 0.01 Da with $\sqrt{2}$-fold change increments, the number of MS1 features ranged from 250 to 8,000 with two-fold change increments, and the number of retention time bins ranged from 1 to 8 with two-fold change increments. The hyperparameter grid for the continuous baseline was the same as the binned baselines except that m/z tolerance (in ppm) was used instead of m/z bin width. The m/z tolerance could take the following values: 1, 4, 16, and 64ppm. The best set of hyperparameters for each binned baseline method can be found in Supplemental Table 4. The best set of hyperparameters for the two continuous baseline can be found in Supplemental Table 5.

2.3 Microbial cultivation

Microbial strains used in this study were: *Escherichia coli* 15597, *Salmonella enterica* serovar Typhimurium 14028, *Bacillus cereus* 14579, *Bacillus thuringiensis* Al Hakam, *Bacillus thuringiensis* HD600 and *Porphyrobacter* sp. HL-46. *Escherichia coli* 15597, *S. enterica* serovar Typhimurium 14028, and *B. cereus* 14579 were cultivated on Tryptic Soy Agar (TSA) from glycerol stocks and then into Tryptic Soy Broth for 16

hyperparameter	hyperparameter range
# of MS1 features	250, 500, 1000, 2000, 4000, 8000
retention time bins	1, 2, 4, 8
m/z tolerance (ppm)	1, 4, 16, 64
m/z bin width (δ_2)	0.0025, 0.0035, 0.005, 0.0071, 0.01

Supplemental Table 3: **Hyperparameter search grid for baselines.** Table of the grid that was searched during the exhaustive hyperparameter search for each of the baselines. Note that m/z bin width is used for the binned baseline while m/z tolerance was used for the continuous baseline. The other two hyperparameters are used by both the binned and continuous baseline.

Method	type	# MS1 features	m/z bin width (Da)	# RT bins (n)
# m/z bins in common	indicator	1000	0.0071	NA
# m/z + RT bins in common	indicator	1000	0.0050	2
# m/z bins in common	count	500	0.0071	NA
# m/z + RT bins in common	count	1000	0.0050	2
# m/z bins in common	min	1000	0.0071	NA
# m/z + RT bins in common	min	1000	0.005	2

Supplemental Table 4: **Best performing binned baseline hyperparameters.** The best performing set of hyperparameters for each binned baseline method. We did not include the best set of hyperparameters for the max version of the baseline as it had poor performance.

Method	# MS1 features	m/z tolerance (ppm)	RT tolerance
continuous base (m/z only)	1000	4	NA
continuous base (m/z + RT)	1000	4	0.5

Supplemental Table 5: **Best performing continuous baseline hyperparameters.** The best performing set of hyperparameters for each continuous baseline method. We did not include the best set of hyperparameters for the max version of the baseline as it had poor performance.

hours at 37 °C. *Bacillus thuringiensis* Al Hakam, and *B. thuringiensis* HD600 were grown at 32 °C in TSB, and *Porphyrobacter* sp. HL-46 was grown in Hot Lake autotroph media as described in Cole et al.[6] Cultures were centrifuged to pellet cellular biomass and washed once in sterile phosphate buffered saline (PBS). PBS was removed and the pellets were stored at -80 °C until prepared for peptide analysis.

2.4 Microbial sample preparation

Bacterial cell pellets were thawed and resuspended in a lysis buffer containing 6 M urea and 14.3 mM betamercaptoethanol in 50 mM Tris-HCl pH 8. Samples were incubated at 60 °C in a thermomixer with gentle shaking (300 rpm) for one hour, then 50 mM ammonium bicarbonate was added to reduce the urea concentration 10 fold. Trypsin Gold (Promega) was resuspended in acetic acid to 1 μg/μL concentration and 2 μL was added to each sample. Samples were incubated overnight at 37 °C in a thermomixer with gentle shaking (300 rpm). Digested peptide samples were spun down briefly to pellet any remaining solid debris and the supernatant was subjected to solid phase extraction (SPE). SPE cartridges (Phenomenex; Strata C18-T (55 μm, 140 Å), 100 mg/1 mL, cat # 8B-S004-EAK) were loaded into the vacuum manifold and samples were processed according to the manufacturers recommendation. Briefly, cartridges were conditioned with 1 mL methanol and washed with 1 mL 0.1% trifluoroacetic acid (TFA) in water. The sample was added to the cartridge, followed by a wash with 1 mL 5% acetonitrile/95% 0.1% TFA in water. Finally, the sample was eluted with 1 mL 80% acetonitrile and 20% 0.1% TFA in water. Samples were concentrated to near dryness down using a SpeedVac and resuspended in 30 μL 0.1% TFA water. A BCA assay (Pierce) was performed to determine peptide concentration. Samples were diluted to 0.1 μg/μL and stored at -20 °C until MS analysis.

2.5 Plant sample processing and preparation

In addition to the bacterial samples used in this study, plant material from *Nicotiana benthamiana* was also used. Leaf plant material was processed and prepared as previously described.[7] Briefly, material was washed, frozen in liquid nitrogen, and ground in a precooled mortar in the presence of liquid nitrogen. Ground plant material (150 mg) was precipitated at -20°C overnight in 10% trichloroacetic acid (TCA) with 0.07% betamercaptoethanol in cold acetone. The next day, the sample was centrifuged at 10,000 × g for 15 minutes, the supernatant was removed, and the remaining pellet was washed twice with ice cold acetone with 0.07% betamercaptoethanol. The pellet was then resuspended in the lysis buffer as described for microbial cell samples and all downstream reduction, denaturation, and digestion steps were the same for the plant sample as for the microbial cell samples.

2.6 Liquid chromatography-tandem mass spectrometry

Lab 1. Liquid chromatography separation was performed using an Agilent 1200 HPLC instrument with a 40 cm long 0.15 mm ID fused silica packed with Jupiter 5 μm C-18 resin. Mobile phase A was prepared with 5% acetonitrile and 0.1% formic acid in nano-pure H2O; mobile phase B was prepared with 95% acetonitrile and 0.1% formic acid in nano-pure H2O. The flow rate was 2 μL per minute with a reversed phase gradient transitioning from 0% solution B to 45% solution B over the course of 60 min for separation followed by a wash and regeneration step. An Orbitrap XL mass spectrometer (Thermo Electron, Thousand Oaks, CA) was used to analyze the eluate from the HPLC, which was directly ionized and transferred into the gas phase with an electrospray emitter (operated at 3.5 kV relative to the mass spectrometer interface). The ion transfer tube on the Orbitrap system was maintained at 200 °C and 200 V with an ion injection time set for automatic gain control with a maximum injection time of 200 ms for 5×10^7 charges in the linear ion trap. Ion selection was achieved using dynamic parent ion selection in which the five most abundant ions were selected for MS/MS using a 3 m/z window. Each sample was analyzed in technical triplicate.
Lab 2. A Waters nano-Acquity dual pumping UPLC system (Milford, MA) was configured for on-line trapping of a 5 μL injection at 5 μL/min with reverse-flow elution onto the analytical column at 300 nL/min. Columns were packed in-house using 360 µm o.d. fused silica (Polymicro Technologies Inc., Phoenix, AZ) with 2-mm sol-gel frits for media retention and contained Jupiter C18 media (Phenomenex, Torrence, CA) in 5μm particle size for the trapping column (150μm i.d. x 4cm long), with 3μm particle size for the analytical column (75 μm i.d. x 70 cm long). Mobile phases consisted of (A) 0.1% formic acid in water and (B) 0.1%

formic acid in acetonitrile with the following gradient profile (min, %B): 0,1; 8,1; 10,8; 28,12; 83,30; 105,45; 108,95; 118,95; 122,50; 124,95; 126,1; 128,50; 130,50; 132,1; 152,1.

MS analysis was performed using an Q Exactive HF mass spectrometer (Thermo Scientific, San Jose, CA) outfitted with a home-made nano-electrospray ionization interface. Electrospray emitters were prepared using 150 μm o.d. x 20 um i.d. chemically etched fused silica.[8] The ion transfer tube temperature and spray voltage were 325°C and 2.3 kV, respectively. Data were collected for 100 min following a 20 min delay from sample injection. FT-MS spectra were acquired from 400-2000 m/z at a resolution of 60k (AGC target 3e6) and while the top 12 FT-HCD-MS/MS spectra were acquired in data dependent mode with an isolation window of 2.0 m/z and at a resolution of 15k (AGC target 1e5) using a normalized collision energy of 30 and a 45 sec exclusion time.

2.7 Database search

A database search was conducted using Crux version 3.2[9,10] on the runs from the multi-species dataset using the combined p-value score function.[11] The spectra were searched against a concatenated database containing the proteomes of the following species: *Porphyrobacter* sp. YT40 (UP000315943), *Salmonella enterica* (UP000054420), *Escherichia coli* (UP000000558), *Bacillus cereus* (UP000001417), *Bacillus thuringiensis* (UP000032057), and *Nicotiana tabacum* (UP000084051). The proteomes were downloaded from Uniprot[12] in April 2021. The protein database was digested into peptides using the tide-index tool, and the search was performed by the tide-search tool. All parameters were set to their default values except that "mz-bin-width" was set to 1.0005079, "score-function" was set to "both", "exact-p-value" was set to "True", and "top-match" was set to one. The false discovery rate was estimated using target-decoy competition[13] and filtered to a 1% PSM level FDR.

The spectra from the *Bacillus* genus data were searched in the same manner as the multi-species dataset except that the spectra were searched against a concatenated database containing the following species: *B. cereus* (UP000001417), *B. cytotoxicus* (UP000002300), *B. mycoides* (UP000001754), *B. pseudomycoides* (UP000001378), *B. thuringiensis* (UP000011719), *B. weihenstephanensis* (UP000002154), and *B. toyonensis* (UP000017860). These proteomes were downloaded from Uniprot[12] in January of 2022.

2.8 Data

Species training dataset. A total of 166 RAW files from 33 PRIDE IDs were downloaded from PRIDE in November of 2020. These 166 runs each originated from a sample that contains one of nine different species (Supplemental Figure 2A). For this dataset we used data from up to five PRIDE IDs per species, except that human had six PRIDE IDs, and downloaded up to five RAW files per PRIDE ID. The mass spectrometry runs were collected using various types of Thermo Scientific instrumentation (San Jose, CA). We did not include any off-line fractionated samples or labeled samples (*e.g.* TMT or ITRAQ). All samples were digested using trypsin. Additional information about this dataset can be found in Supplemental File 1.

Species test dataset. An additional 130 RAW files from 23 PRIDE IDs were downloaded from PRIDE in December of 2020 for compilation into a species test dataset. Each run consists of data from one of nine different species (Supplemental Figure 2B). Like the training dataset, we used five RAW files per PRIDE ID; however, we only used data from three different PRIDE IDs per species. One exception is that we only collected *Plasmodium falciparum* data from two different PRIDE IDs. The other exception is that all 15 runs of the *Yersinia pestis* data came from a single PRIDE ID. However, the data from that project itself is an aggregation of several different experiments. None of the PRIDE IDs nor runs in this dataset overlap with the data in the species training dataset. Additional information about this dataset can be found in Supplemental File 2.

Multi-species data. This dataset consists of 16 different samples (labeled A-P) that were each injected into a mass spectrometer four times, which resulted in 63 runs (sample P was run three times). Each sample contains up to three different organisms from a set of seven possible species (Supplemental Table 7). If a sample contained multiple species then they were mixed together in equal concentrations. Each sample was injected into an LTQ-Orbitrap XL three times and one time into an Q-Exactive. We note that the LTQ-Orbitrap XL and the Q-Exactive instruments are from different laboratories and therefore have different liquid chromatographies and instrument parameters. Additional information about this dataset

can be found Supplemental File 3, and the raw files have been deposited into PRIDE with dataset identifier PXD027791.

***Bacillus* genus data.** A total of 42 RAW files were downloaded from PRIDE (PXD003669) in November of 2021.[14] These runs originated from seven different strains of the *Bacillus cereus* group. The seven strains analyzed were *B. cereus* DSM 31, *B. cytotoxicus* DSM 22905, *B. mycoides* DSM 2048, *B. pseudomycoides* DSM 12442, *B. thuringiensis* DSM 2046, *B. weihenstephanensis* DSM 11821, and *B. toyonensis* CECT 876. A total of six runs, two technical replicates of three independent biological replicates, for each strain was run on a nanoAcquity UPLC (Waters Inc., Milford, USA) connected to a Q-Exactive mass spectrometer (Thermo Scientific). Additional information about this dataset can be found Supplemental File 4.

3 Note

3.1 Explanation why supermodular methods outperform modular methods

Our work showed evidence that supermodular methods outperform modular methods. Here we speculate why modular terms may be insufficient for measuring similarities between mass spectrometry runs by discussing the modular M_3 term. A single edge will score highly with M_3 when the normalized retention time between the two features that make up an edge are very similar. At first glance, this seems reasonable because two peptide features with the same m/z and retention time should be the same ion. However, this observation fails to account for systematic differences in retention time that result from differing liquid chromatography conditions. Therefore, while we expect the order in which peptides elute off the column to approximately match between two runs, we do not expect such correspondence in retention time. As a result, an edge with a large retention time shift could be correct. Using supermodularity to consider pairs of edges overcomes this issue because it considers a neighborhood of edges. If a set of edges in the same region have the same retention time shift, it indicates that a systemic time shift has occurred and the retention time shifts are correct.

Timing of MS1Connect

To gauge the speed of MS1Connect, we measured how long it took to calculate all of the MS1Connect scores for the species test set. This dataset consists of 130 runs, which results in 8,515 MS1Connect scores. The entire analysis took 372.71 hours on one Intel Xeon E5-2820 CPU. Although this is a significant amount of time, we note that MS1 feature detection is by far the most time intensive step. MS1 feature detection, which was performed on 130 runs, took 316.31 (96.49%) hours. On the other hand, the remaining steps, which were performed on 8,851 pairwise runs, only took 11.5 (3.51%) hours. We note that the MS1 feature detection needs only to be performed once per run since the output can be stored. In addition, we note that every step can be sped up using multiple threads in an embarrassingly parallel fashion.

4 Description of Supplemental Files

- **supplemental-file-1.txt** This file contains metadata information for all the runs found in the species training set. Data in this file was used to generate Figure 2.
- **supplemental-file-2.txt** This file contains metadata information for all the runs found in the species test set. Data in this file was used to generate Supplemental Figure 5.
- **supplemental-file-3.txt** This file contains metadata information for all the runs used to generate Figure 3.
- **supplemental-file-3.txt** This file contains metadata information for all the runs used to generate Figure 4.

Variable	Definition
$G = (U, V, E)$	Bipartite graph containing two vertex sets U and V and edge set E
U	vertex set of MS1 features from one run
V	vertex set of MS1 features from a second run
r	mass spectrometry run
E	set of valid edges
u	node in U
v	node in V
e	instance of valid edge
$s(e)$	normalized retention time shift of edge e
$u(e)$	MS1 feature u that is a part of edge e
$v(e)$	MS1 feature v that is a part of edge e
$t(u)$	normalized retention time of MS1 feature u
$m(u)$	m/z of MS1 feature u
$I(u)$	normalized intensity of u
$c(u)$	charge of MS1 feature u
A	set of edges scored by objective function
S	objective function used to a score a set of edges
$\mathcal{E}_V$	degree constraint on nodes in V
$\mathcal{E}_U$	degree constraint on nodes in U
δ_1	m/z tolerance in ppm
δ_2	m/z bin width in Daltons
N	the number of most intense MS1 features used to represent a run
n	number of retention time bins
M_i	one of four terms in the objective function
λ_i	hyperparameter that weights the importance of each of the four terms in the objective function
α	hyperparameter that is used in M_3
β	hyperparameter that is used in M_4
γ	hyperparameter that is used in M_4
κ^f	supermodular curvature
P_k	precision at k
R_k	recall at k
q	query run
R	set of repository runs
C	number of repository runs
X	matrix representation of mass spectrometry run
Y	matrix representation of mass spectrometry run
G	function that is either the product, maximum, minimum, or indicator of two input values

Supplemental Table 6: **Notation.** Notation used in this manuscript.

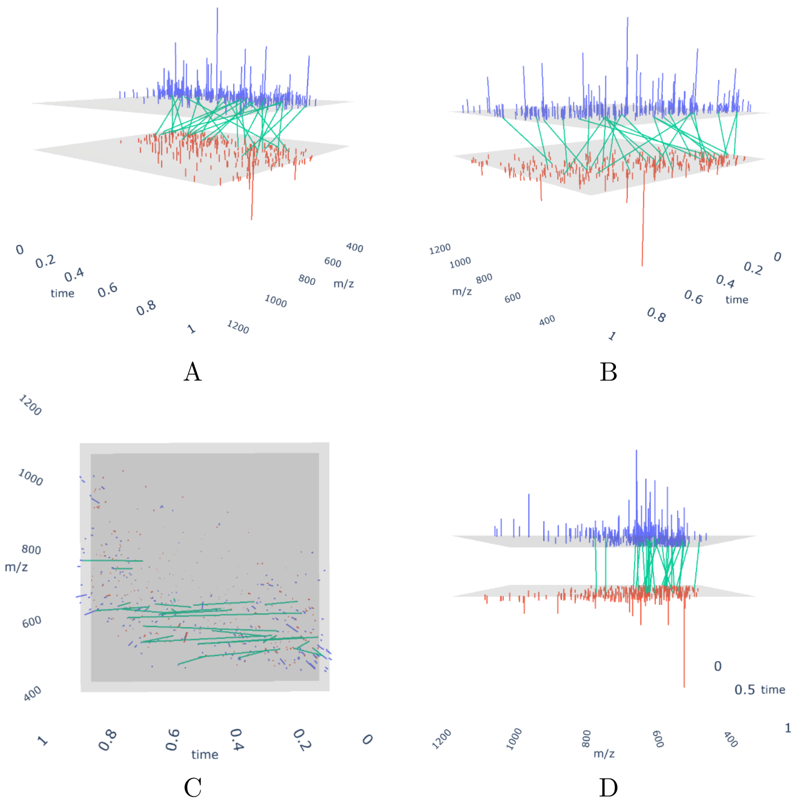

Supplemental Figure 1: **Views of bipartite graph.** Four views of the bipartite graph formed by a pair of mass spectrometry runs. The purples lines are the MS1 features from one run (006_EC-D2O_A17.raw) and the orange lines are the MS1 features from the second run (11B_RINICHI_sTgw10.raw). The green lines represent the set of all possible edges E that link MS1 features with similar m/z. The three axes shown in these plots are m/z retention time, and intensity. The two gray planes represent the bottom of each run, with respect to intensity. Note in D how edges are nearly vertical and only link MS1 features with similar m/z.

Sample ID	Porphyrobacter	*S. enterica*	*E. coli*	*B. cereus*	*B. thuringiensis* Al Hakam	*B. thuringiensis* HD600	*N. benthamiana*
A	✓						
B		✓		✓			
C			✓				
D				✓			
E					✓		✓
F						✓	
G	✓				✓	✓	
H			✓	✓			✓
I	✓						✓
J	✓		✓				
K		✓					
L					✓	✓	
M					✓		
N				✓		✓	
O		✓	✓				
P							✓

Supplemental Table 7: **Table of species data.** A table of the species found in each sample. Note that *B. cereus* is *Bacillus cereus* 14579, *S. enterica* is *Salmonella enterica* Typhimurium strain 14028, and *E. coli* is *Escherichia coli* 15597. In addition, *B. cereus*, *B. thuringiensis* Al Hakam, and *B. thuringiensis* HD600 are often considered the same species. Horizontal lines are used visualization purposes.

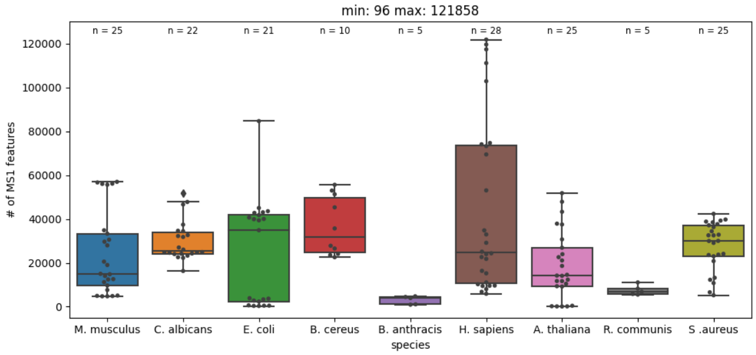

A

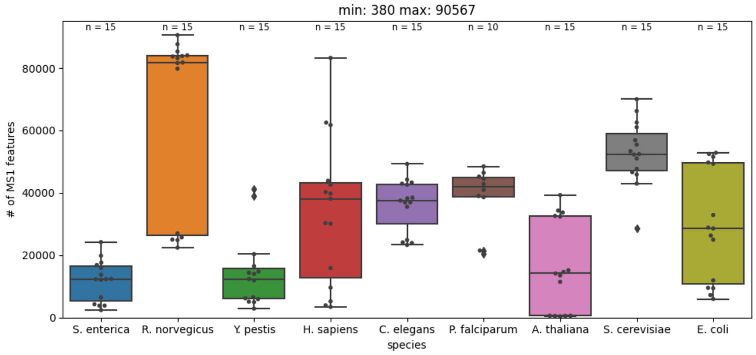

B

Supplemental Figure 2: **Number of MS1 features per run.** A series of boxplots of the number of MS1 features per run split by species for the (A) species training data and (B) the species test data. In addition to the boxplots, each point shows the number of MS1 features per run. The text near the top of the plot indicates the number of runs that each label has.

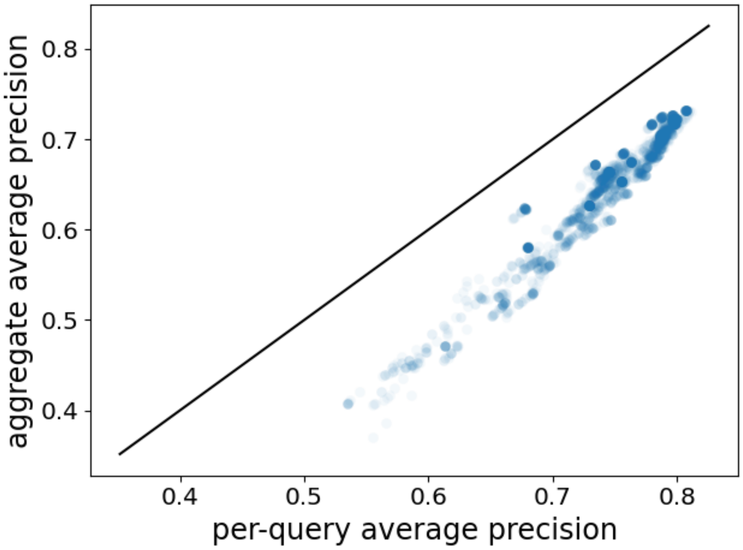

Supplemental Figure 3: **Comparison of per-query average precision and aggregate average precisio.** Scatter plot of the per-query average precision and aggregate average precision of the species training dataset over 2,500 different hyperparameterizations. These metrics are highly correlated with a Pearson correlation of 0.9689. The solid black line shows the $y = x$ line.

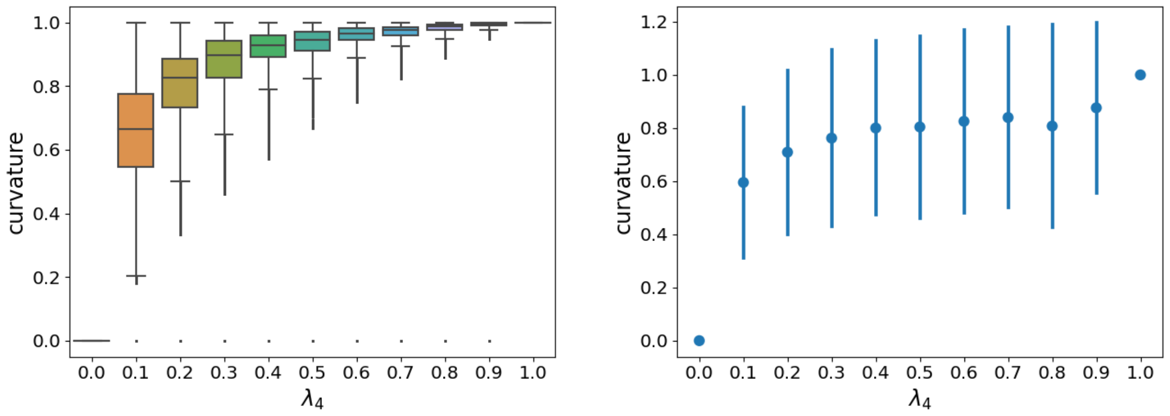

A B

Supplemental Figure 4: **Curvature as a function of λ_4.** These plots show the curvature value as a function of λ_4. The best performance of MS1Connect occurs when $\lambda_4 = 0.9$ A) A boxplot of the curvature values showing mean and inter-quartile range. B) A plot of the mean and standard deviation of the curvature values. Note that curvature values, by definition, range from zero to one, inclusive.

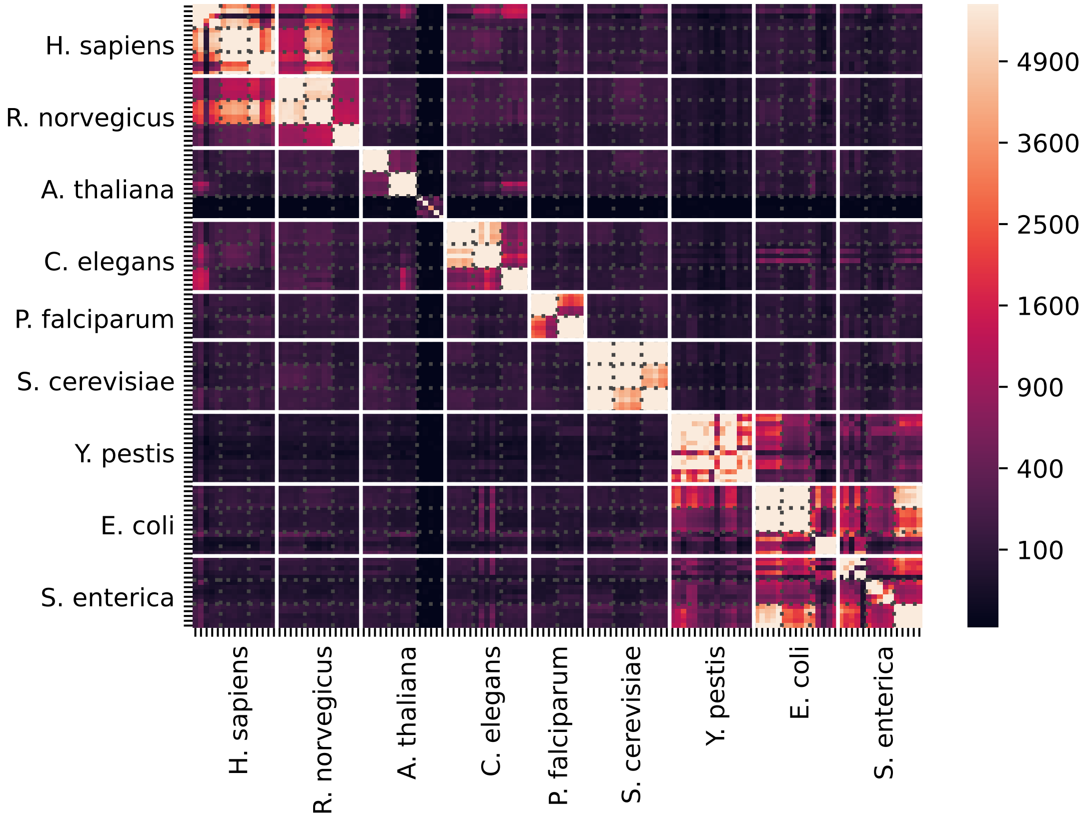

Supplemental Figure 5: **Heatmap of MS1Connect scores for species test data.** Each cell is colored by the MS1Connect score between a pair of runs. The solid white lines denote the border between different species while the dotted gray lines delineate the border between different experiments (PRIDE ID).

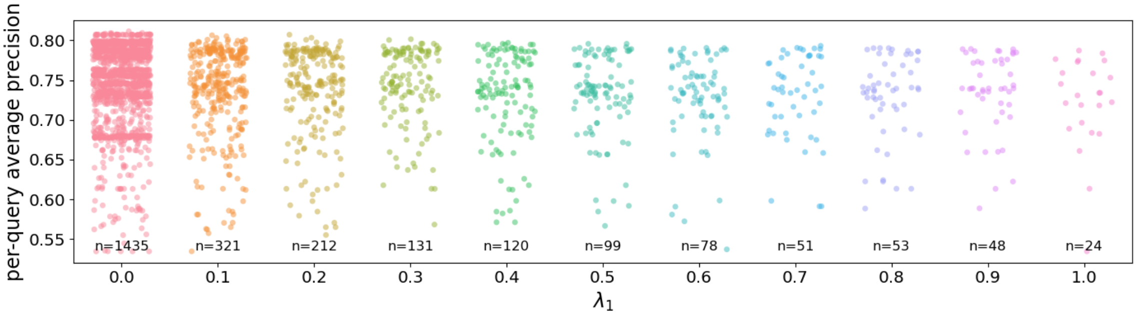

A

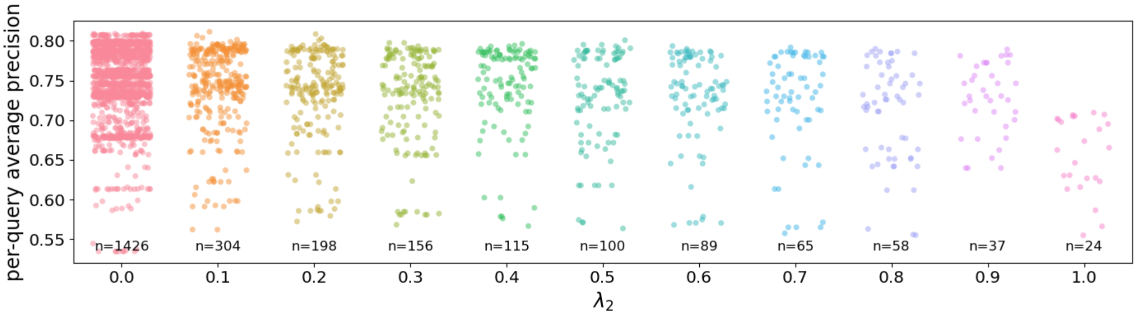

B

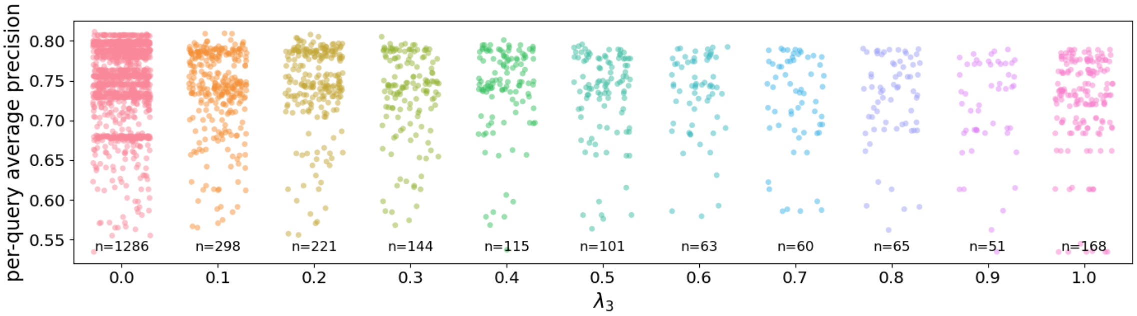

C

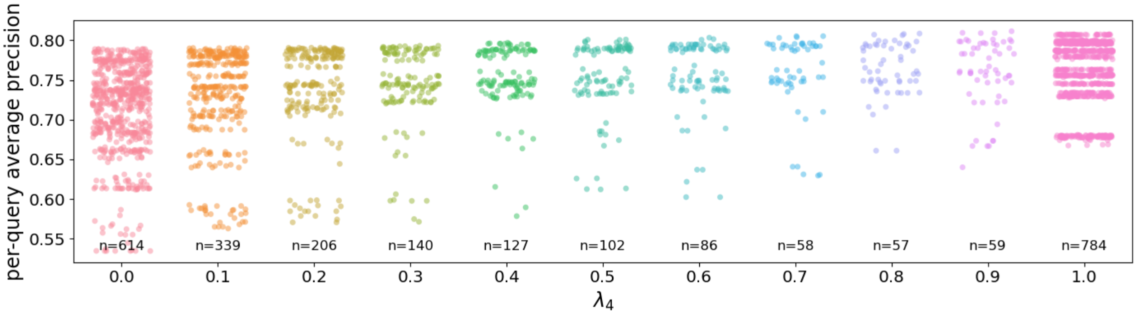

D

Supplemental Figure 6: **Performance as a function of λ.** This figures plots the performance of 2500 different hyperparameterizations of MS1Connect as a function of (A) λ_1, (B) λ_2, (C) λ_3, (D) or λ_4. Note that the values near the bottom are the number of points found in each column.

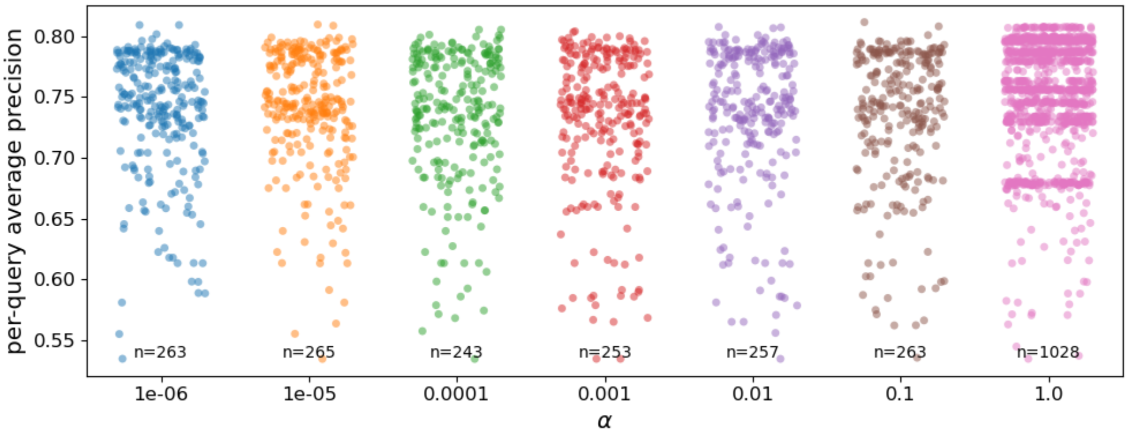

A

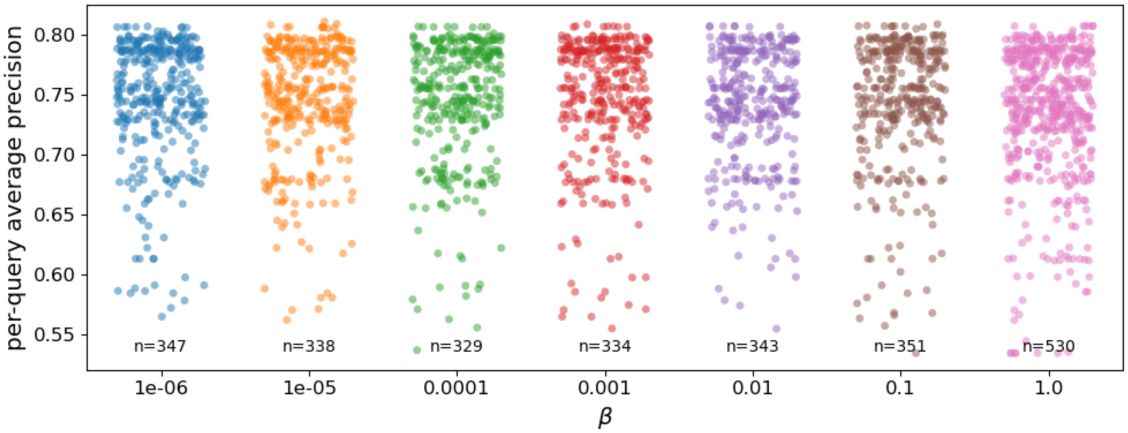

B

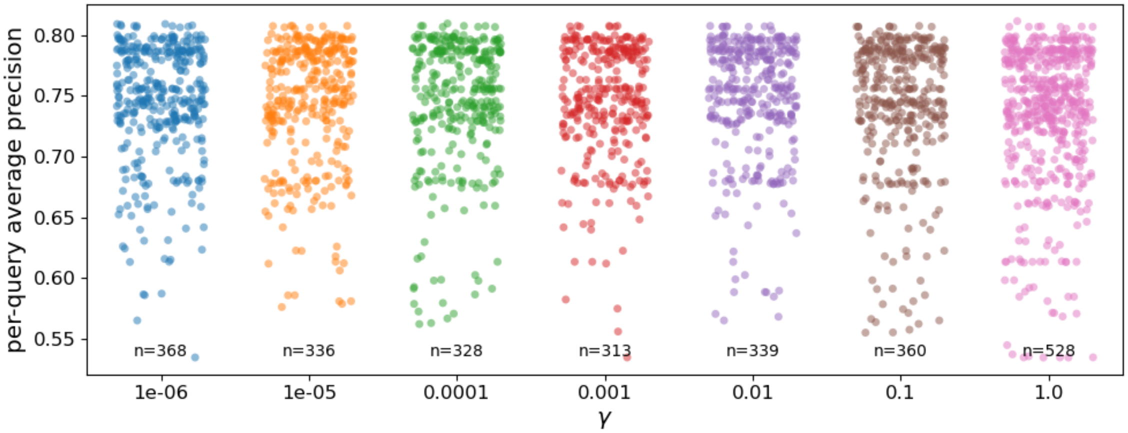

C

Supplemental Figure 7: **Performance as a function of α, β, or γ.** This figures plots the performance of 2500 different hyperparameterizations of MS1Connect as a function of (A) α, (B) β, (C) or γ. Note that the values near the bottom are the number of points found in each column.

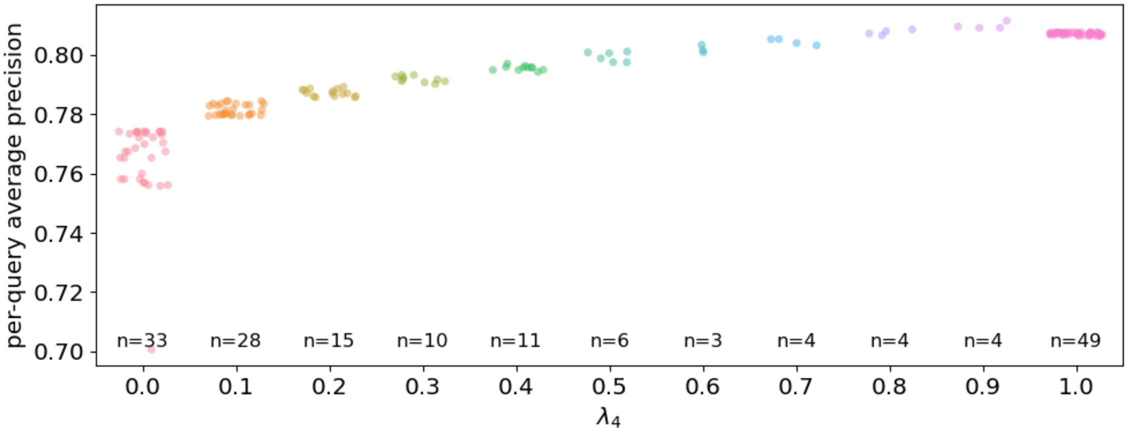

A

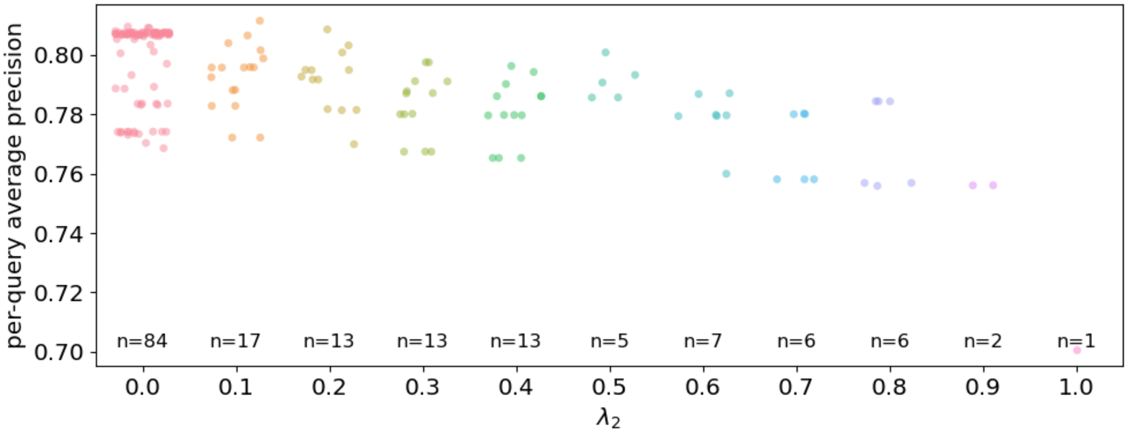

B

Supplemental Figure 8: **Performance of various MS1Connect hyperparameterizations after fixing N and δ_1.** Each panel plots plots the performance of MS1Connect as a function of (A) λ_4 or (B) λ_2. Performance tends to decrease as λ_2 increases from 0.0 to 1.0. In addition, performance tends to increase as λ_4 increases from 0.0 to 0.9 and then decreases as λ_4 increases from 0.9 to 1.0. Note that the values near the bottom are the number of points found in each column. For both plots, there tends to be fewer points as the λ value increases because $\sum_1^4 \lambda_i = 1.0$. In addition, there are fewer points when λ_2 is large because M_2 does not use hyperparameters α, β, and γ.

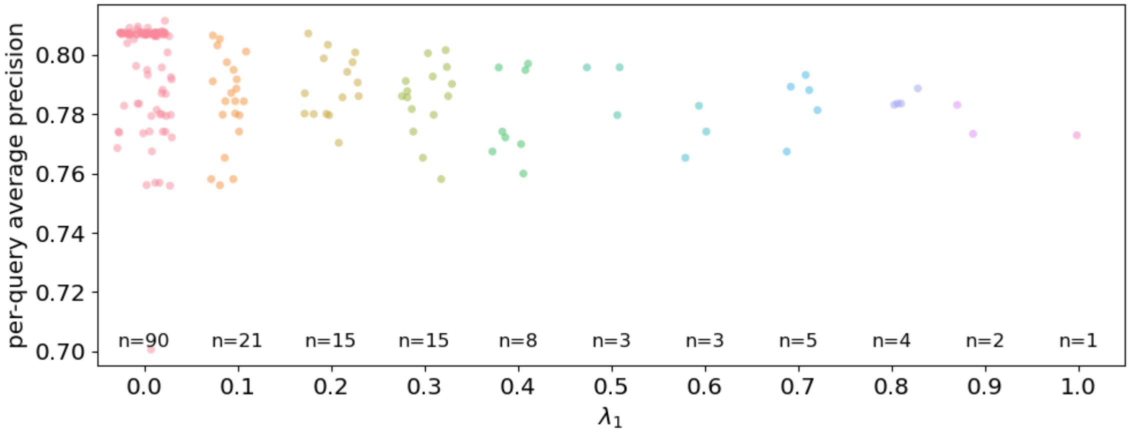

A

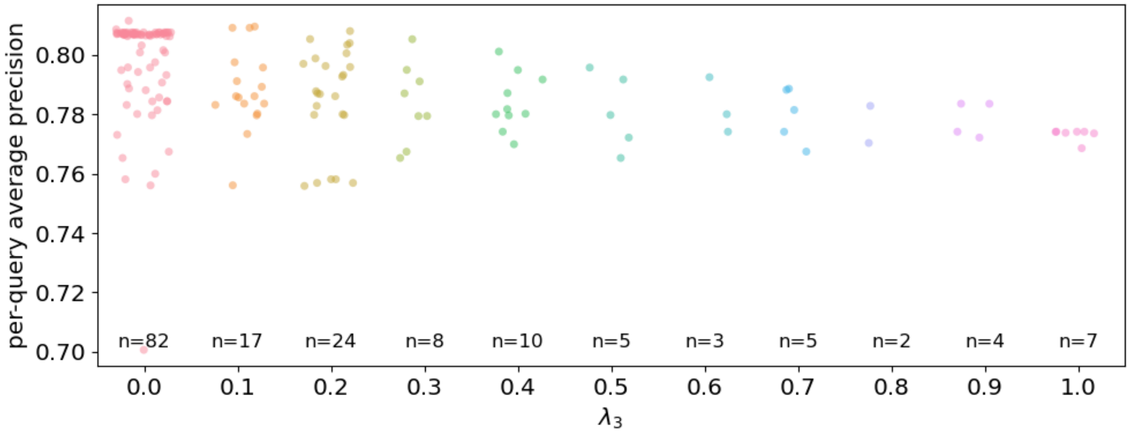

B

Supplemental Figure 9: **Performance as a function of λ_1 or λ_3.** This figures plots the performance of MS1Connect, given $N = 4000$ and $\delta_1 = 4$ ppm, as a function of (A) λ_1 (B) or λ_3. Note that the values near the bottom are the number of points found in each column. For both plots, there tends to be fewer points as the λ value increases because $\sum_1^4 \lambda_i = 1.0$.

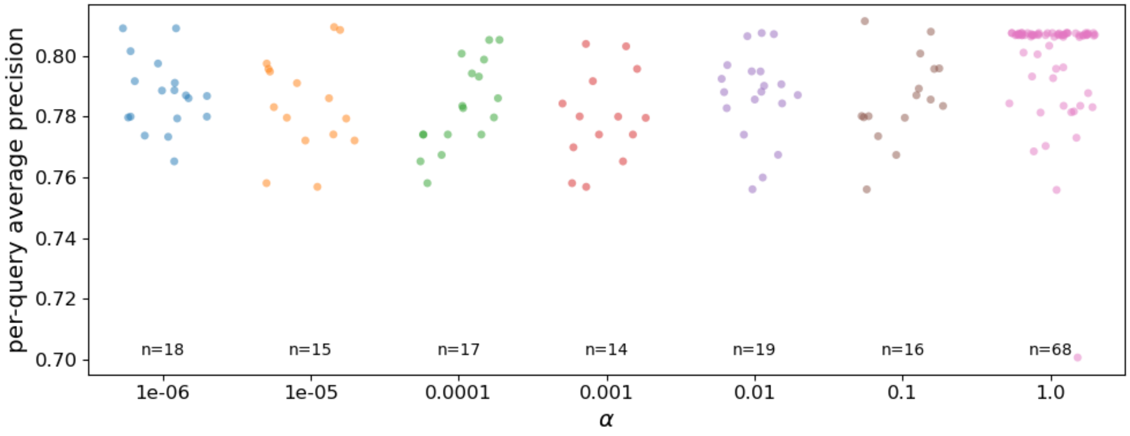

A

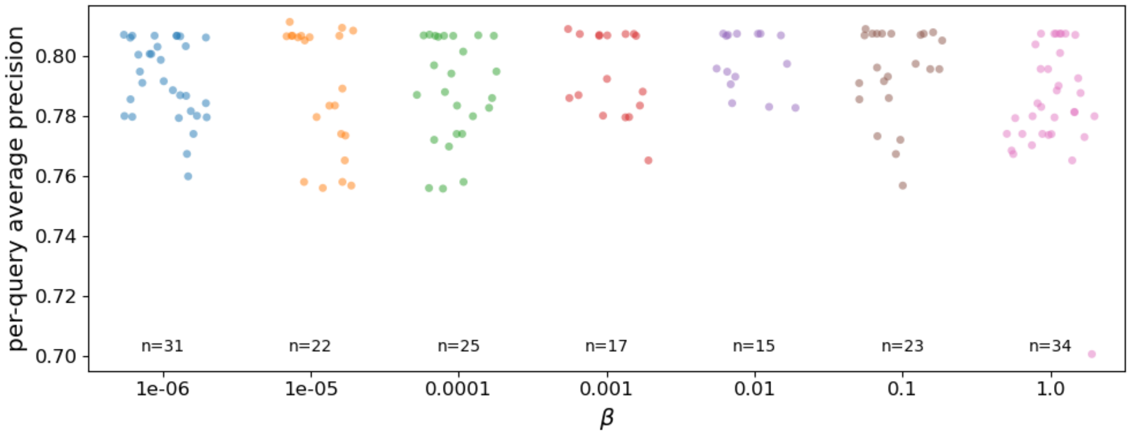

B

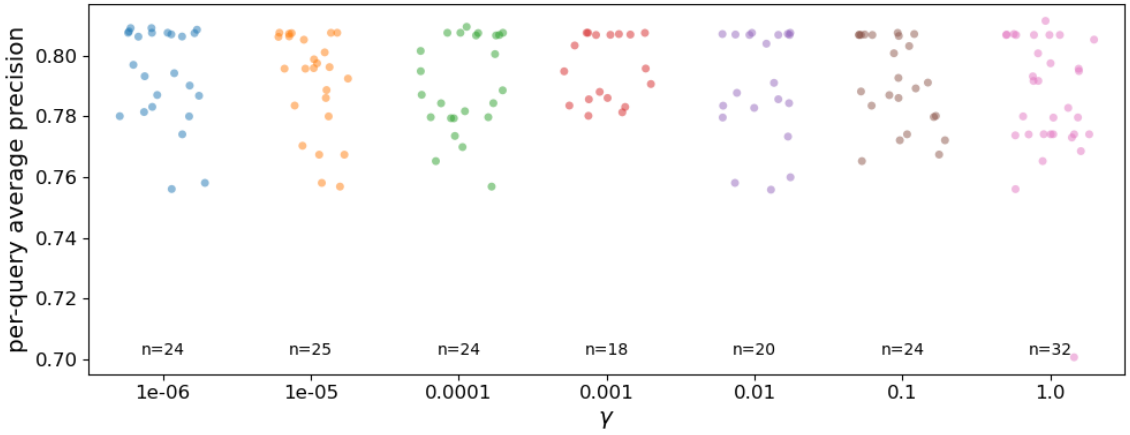

C

Supplemental Figure 10: **Performance as a function of α, β, or γ.** This figures plots the performance of MS1Connect, given $N = 4000$ and $\delta_1 = 4$ ppm, as a function of (A) α, (B) β, (C) or γ. In general, the values of α, β, and γ are not correlated with performance. Note that the values near the bottom are the number of points found in each column.

References

[1] J. Edmonds. Matroids, submodular functions, and certain polyhedra. *Combinatorial Structures and Their Applications*, pages 69–87, 1970.

[2] J.G. Oxley. *Matroid Theory: Second Edition.* Oxford University Press, 2011.

[3] U. Feige. A threshold of *ln n* for approximating set cover. *Journal of the ACM*, 45(4):634–652, 1998.

[4] H. Lin and J. A. Bilmes. Optimal selection of limited vocabulary speech corpora. In *Proc. Annual Conference of the International Speech Communication Association (INTERSPEECH)*, Florence, Italy, August 2011.

[5] Wenruo Bai and Jeff Bilmes. Greed is still good: maximizing monotone submodular+ supermodular (bp) functions. In *International Conference on Machine Learning*, pages 304–313. PMLR, 2018.

[6] J. K. Cole, J. R. Hutchison, R. S. Renslow, Y. M. Kim, W. B. Chrisler, H. E. Engelmann, A. C. Dohnalkova, D. Hu, T. O. Metz, J. K. Fredrickson, and S. R. Lindemann. Phototrophic biofilm assembly in microbial-mat-derived unicyanobacterial consortia: model systems for the study of autotroph-heterotroph interactions. *Front Microbiol*, 5:109, 2014.

[7] M. Chatterjee, S. Gupta, A. Bhar, and S. Das. Optimization of an Efficient Protein Extraction Protocol Compatible with Two-Dimensional Electrophoresis and Mass Spectrometry from Recalcitrant Phenolic Rich Roots of Chickpea (Cicer arietinum L.). *Int J Proteomics*, 2012:536963, 2012.

[8] R. T. Kelly, J. S. Page, Q. Luo, R. J. Moore, D. J. Orton, K. Tang, and R. D. Smith. Chemically etched open tubular and monolithic emitters for nanoelectrospray ionization mass spectrometry. *Analytical Chemistry*, 78(22):7796–7801, Nov 2006.

[9] S. McIlwain, K. Tamura, A. Kertesz-Farkas, C. E. Grant, B. Diament, B. Frewen, J. J. Howbert, M. R. Hoopmann, L. Käll, J. K. Eng, M. J. MacCoss, and W. S. Noble. Crux: rapid open source protein tandem mass spectrometry analysis. *Journal of Proteome Research*, 13(10):4488–4491, 2014.

[10] C. Y. Park, A. A. Klammer, L. Käll, M. P. MacCoss, and W. S. Noble. Rapid and accurate peptide identification from tandem mass spectra. *Journal of Proteome Research*, 7(7):3022–3027, 2008.

[11] A. Lin, J. J. Howbert, and W. S. Noble. Combining high-resolution and exact calibration to boost statistical power: A well-calibrated score function for high-resolution MS2 data. *Journal of Proteome Research*, 17:3644–3656, 2018.

[12] The UniProt Consortium. UniProt: a worldwide hub for protein knowledge. *Nucleic Acids Research*, pages D506–D515, 2019.

[13] J. E. Elias and S. P. Gygi. Target-decoy search strategy for increased confidence in large-scale protein identifications by mass spectrometry. *Nature Methods*, 4(3):207–214, 2007.

[14] S. Pfrunder, J. Grossmann, P. Hunziker, R. Brunisholz, M. Gekenidis, and D. Drissner. Bacillus cereus group-type strain-specific diagnostic peptides. *Journal of Proteome Research*, 15(9):3098–3107, September 2016.
